# Quackery in England

**Published:** 1844-08

**Authors:** 


*Quackery in England.*—Quackery, like sin, is very ancient. It flourished in ancient Rome, as well as in modern Europe. Nor does it depend for its prosperity on the ignorance of the uneducated classes. The faith of that singular compound of folly and knavery, the world, is kept up by peers, judges, and bishops, by clowns, operatives, and old women, who furnish certificates to the value of nostrums, and testify in favor of impostures, delusion, and villiany. In this country the sale of quack medicines has kept pace with the 'march of intellect.' Forty years ago they yielded an anual revenue to the State of about £14,000. In 1841 the amount received was £50,000. For the last half century, English governments have looked upon this vile revenue as more valuable, in their judgment, than the health of the people, the prosperity of the regular profession, and the improvement of physic.—*London Lancet.*

